# Virus ecology and disturbances: impact of environmental disruption on the viruses of microorganisms

**DOI:** 10.3389/fmicb.2014.00700

**Published:** 2014-12-12

**Authors:** Heather K. Allen, Stephen T. Abedon

**Affiliations:** ^1^National Animal Disease Center, Agricultural Research Service, United States Department of AgricultureAmes, IA, USA; ^2^Department of Microbiology, The Ohio State UniversityMansfield, OH, USA

**Keywords:** bacteriophages, environmental disturbance, phage ecology, aquatic microbiology, phage therapy, metaviromes, evolution, microarrays

Viruses of microorganisms (VoMs)—the encapsidated, acelluluar parasites of archaea, bacteria, and microbial eukaryotes (Hyman and Abedon, 2012)—can be both responsive to and the source of change in their environments. Defining the diverse VoMs in an environment has become easier with the advent of various new technologies, so analyzing VoM populations or communities involved with disturbances could be the next step to drive research forward. Contributions assembled in this Research Topic explore the effects of disturbances on one type of VoM, the bacteriophages (phages), as well as disturbances caused by phages. The collection presented in this ebook opens with an editorial describing the importance of change to life on Earth, and by defining the multiple levels at which change can occur: genetic, organismal, and environmental (Allen and Abedon, 2013). We have arranged and introduced the manuscripts in this collection based on which of these levels they address.

## Genetic change

Organismal responses to certain disturbances can be tightly linked to evolution. Two papers in this Research Topic contribute to evolutionary theory using two entirely different approaches: RNA virus populations *in vitro* and viral metagenomes *in silico*. Goldhill et al. studied the effect of repeated heat stress on an RNA virus to test the relationship between the maintenance of phenotype despite ongoing genetic mutations, which is called robustness, and the heritable ability to adapt to environmental disturbances, or evolvability (Goldhill et al., 2014). Theirs is one of the first studies to investigate these questions in the laboratory, using the ϕ6 RNA phage of *Pseudomonas syringae* pv. *phaseolicola*. The contribution by Mizuno et al. examines marine viral metagenomes for metaviromic islands, which are gaps in the metagenome represented by significantly fewer sequences than neighboring regions of high coverage (Mizuno et al., 2014). They show that the metaviromic islands frequently correspond to genes involved in genome packaging, molecular recognition, and host recognition, the latter of which is suggestive of providing high diversity within lineages to maximize predatory opportunities.

## Organismal change

Next are two papers that use examples from agriculture to consider the relationship between phages and disturbances at the organismal level. Bearson et al. show that subinhibitory concentrations of the agricultural antibiotic carbadox induce prophages *in vitro* in the foodborne pathogen *Salmonella* Typhimurium (Bearson et al., 2014). Additional data demonstrate phage-mediated gene transfer by phages induced by this antibiotic. The piece by Meaden and Koskella is a review on phage therapy, with an emphasis on agricultural examples (Meaden and Koskella, 2013). Phage therapy is a potential alternative to antibiotic therapy, and the authors thoughtfully explore the potential risks and unknowns associated with phage therapy.

## Environmental change

The effect of environmental change on viruses is explored here in aquatic ecosystems. Storms are a powerful macro-scale disturbance to any system, and Williamson et al. examines the effect of stormwater runoff and the viruses therein on freshwater microbial communities (Williamson et al., 2014). Unique to this study is consideration of both the planktonic and particle-associated viral communities. Moving to a marine system, Labonté and Suttle undertake a deep analysis of the *Gokushovirinae* family of single-stranded DNA viruses to assess their diversity on a nearly global scale (Labonté and Suttle, 2013). The lineages studied showed environment-dependent biogeographic distributions.

A special type of environmental change can occur in host-associated ecosystems, where interaction with hosts adds an additional layer of complexity. The work by Payet et al. continues the aquatic theme with an analysis of viral diversity in coral reef aquatic ecosystems (Payet et al., 2014). They undertook an extended spatiotemporal analysis of viral abundance and lytic activity in the South Pacific Ocean, with the results revealing viral dynamism linked to microbial communities and environmental factors. Next, Yamada presents a hypothesis on the regulation of virulence by filamentous phages of *Ralstonia*, which is the causative agent of bacterial wilt disease in certain agricultural plants (Yamada, 2013). The author suggests a phage-encoded open reading frame that is responsible for mediating virulence. Finally, Santos et al. review the potential for microarrays to dissect viral-microbe interactions, which could be applied to samples from any environment (Santos et al., 2014). The use of various probes, including glycans, peptides, and nucleic acids, provides a powerful platform for investigating a wide range of questions in viral ecology.

## Concluding remarks

The articles in this Research Topic uniquely and individually address one of the tiniest biological entities on Earth, the viruses of microorganisms, and the enormous role they play in both causing and effecting environmental change. This collection also provides insights into new techniques and approaches that will be valuable to furthering research on viral ecology.

### Conflict of interest statement

Heather K. Allen declares that the editing of this volume was conducted in the absence of any commercial or financial relationships that could be construed as a potential conflict of interest. Stephen T. Abedon undertakes paid consulting for phage therapy-associated businesses but was not involved in editing the phage therapy-associated chapter in this volume (Meaden and Koskella, 2013).
